# Editorial: Childhood Rickets—New Developments in Epidemiology, Prevention, and Treatment

**DOI:** 10.3389/fendo.2020.621734

**Published:** 2020-11-23

**Authors:** Peter J. Simm, Craig F. Munns, Craig Alan Jefferies, Benjamin John Wheeler

**Affiliations:** ^1^ Department of Endocrinology and Diabetes, Royal Children’s Hospital, Melbourne, VIC, Australia; ^2^ Murdoch Children’s Research Institute, Melbourne, VIC, Australia; ^3^ Department of Paediatrics, University of Melbourne, Melbourne, VIC, Australia; ^4^ Institute of Endocrinology & Diabetes, Children’s Hospital Westmead, Sydney, NSW, Australia; ^5^ Discipline of Child & Adolescent Health, University of Sydney, Sydney, NSW, Australia; ^6^ Paediatric Endocrinology, Starship Children’s Health, Auckland, New Zealand; ^7^ Liggins Institute, University of Auckland, Auckland, New Zealand; ^8^ Department of Women’s and Children’s Health, Dunedin School of Medicine, University of Otago, Dunedin, New Zealand; ^9^ Paediatric Department, Southern District Health Board, Dunedin, New Zealand

**Keywords:** rickets, vitamin D, calcium, phosphate, growth

Despite being first described over 300 years ago, rickets remains an ongoing important public health concern. Rickets remains a significant and preventable cause of global morbidity and, sadly, even mortality. The most common form, nutritional rickets, is still widespread, and there are vast numbers of ongoing cases across the world (1). While our understanding of rickets has advanced significantly in terms of underlying causes, pathophysiology and treatment, translating this knowledge into practical community health programs to work towards the elimination of nutritional rickets remain underdeveloped and ineffective. While far less common, inherited causes of rickets, relating to dysregulation of phosphate and/or calcium metabolism, are often missed, leading to inappropriate therapies and poorer clinical outcomes (2).

The contributors to this Research Topic have demonstrated varied approaches to considering the issue of rickets. Hochberg and Hochberg took an evolutionary approach, reflecting on the strongly conserved nature of the vitamin D receptor gene from phytoplankton to mammals, the changes in skin pigmentation with historical migration, and the natural selection disadvantages in those with severe Vitamin D deficiency, both for childhood rickets sufferers (with marked disability and reduced survival) and in pregnancy. Lessons for today’s society conclude their thought provoking article, including the role that technology and modern lifestyles play in reducing our sunlight exposure and thus exposing us to greater risk of Vitamin D deficiency. This article was penned before COVID-19, but given the number of countries across the globe that have used lockdowns and prolonged periods of home isolation to help reduce the spread of the novel coronavirus, it is even more apt to now consider how important adequate supplementation is for prevention, and how lifestyle factors play a vital part of in our risk of developing Vitamin D deficiency.

Wheeler et al. also chose to focus on the historical perspective of rickets, and how despite centuries of knowledge, nutritional rickets continues to be seen in the 21^st^ century. While rickets was first described as a clinical entity in the 17^th^ century, there are references to rickets in medical writings from Ancient Greek and Roman times, and skeletal changes consistent with rickets seen in skeletons from pre-industrial Europe. It is the spike in rickets seen with industrialization that provides the greatest lessons for us today, with overcrowding, increased time indoors, poor air quality and reduced calcium intake likely contributing greatly to the increase in presentations at that time. With the discovery of Vitamin D and the effectiveness of cod liver oil in the early 20^th^ century, we now had a means of treating nutritional rickets.

Wheeler et al. conclude by drawing parallels between eras, with many of the conditions that drove rickets in previous centuries echoed in today’s society, such as: reduced exposure to sunlight, the end result of increased time spent indoors; protection from the sun due to valid concerns about the risk of skin cancers; and increased migration of those with deeper skin pigmentation to subtropical and temperate climes. As with the Hochberg’s article, by exploring the mirroring of today’s risk factors with those from the past, hopefully it will encourage us as clinicians to drive public health campaigns to counteract modern changes in lifestyle with increased fortification of foodstuffs with Vitamin D, and ensuring adequate supplementation in high risk groups, particularly at times of high demand for Vitamin D such as pregnancy and infancy (especially for those that are breast fed).

By contrast, Michigami and Ozono explored the fundamental molecule implicated in rickets by examining the roles of phosphate in the skeleton. They discussed the number of different ways that hypophosphatemia may be involved in the pathophysiology of rickets. Most directly, phosphate is required for the formation of hydroxyapatite, the basis of mineralization. However, phosphate is also required for apoptosis of hypertrophic chondrocytes in the growth plate, and without this there is disorganization of the process of endochondral ossification, and therefore development of rickets. Finally, the role of phosphate in regulation of gene expression was discussed, including the intriguing finding that phosphate has a role in promoting FGFR transcription, which in turn leads to increased production of FGF23. Therefore, it may be that, in some forms of hypophosphatemic rickets, impaired phosphate sensing may be driving the clinical phenotype.

Finally in this series, Nicolescu et al. provided some clinical perspective, highlighting that even with rare forms of rickets, novel therapeutics are increasingly available to help ameliorate the biochemical, and hopefully in future, the clinical features of these conditions. They describe a case of Vitamin D resistant rickets with a heterozygous mutation in the VDR gene, and outline the treatment challenges with conventional therapy of high dose calcium and calcitriol replacement. They also note previous case reports where the use of cinacalcet, a calcimimetic, has been successful, as the skeletal changes are driven at least in part by the secondary hyperparathyroidism that results from reduced gut absorption and subsequent low serum calcium. They chose a strategy of intravenous calcium absorption, and then added cinacalcet once the calcium levels had stabilized. This led to a normalization of PTH, calcium, phosphate and ALP levels, with improved clinical state (able to ambulate) and resolution of rachitic features on Xray. Clearly there is a need for more systematic study of this approach, which given the rare nature of this condition will require the collaboration of many specialist Paediatric bone centres. This approach will also allow exploration of concerns over the use of cincalcet on the QT interval. In general, for rare bone disorders, it is only by undertaking multicentre studies that we will be able to properly evaluate novel management strategies and assess both their efficacy and safety.

Overall, our topic demonstrates that rickets remains an active, if not growing, area of clinical need. Promotion of public health strategies for prevention of nutritional rickets remains essential, and learning from the lessons of the past will help us improve the future for many children across the globe. Improved molecular understanding of the role of crucial molecules like phosphate will hopefully led to novel therapeutic approaches, such as we have seen with the recent development of monoclonal antibody therapy for X-Linked hypophosphatemic rickets (XLH). Finally, only by collaborating across sites and countries can we explore the outcomes of novel therapeutics in rare hereditary forms of rickets. Hopefully this Research Topic will stimulate us all, as clinicians and/or researchers, to continue to strive to eliminate nutritional rickets and improve outcomes in hereditary rickets, for the benefit of millions of young people around the world.

## Author Contributions

All authors conceived the manuscript. PS wrote the preliminary draft and all authors were involved in its editing. All authors contributed to the article and approved the submitted version.

## Conflict of Interest

CM and PS are members of the advisory board for Kyowa Kirin and have received speaker fees.

The remaining authors declare that the research was conducted in the absence of any commercial or financial relationships that could be construed as a potential conflict of interest.
